# The mosquito melanization response requires hierarchical activation of non-catalytic clip domain serine protease homologs

**DOI:** 10.1371/journal.ppat.1008194

**Published:** 2019-11-25

**Authors:** Layla El Moussawi, Johnny Nakhleh, Layla Kamareddine, Mike A. Osta

**Affiliations:** 1 Department of Biology, American University of Beirut, Beirut, Lebanon; 2 Department of Biomedical Sciences, Qatar University, Doha, Qatar; Johns Hopkins University, Bloomberg School of Public Health, UNITED STATES

## Abstract

Serine protease cascades regulate important insect immune responses namely melanization and Toll pathway activation. An important component of these cascades are clip-domain serine protease homologs (cSPHs), which are non-catalytic, but essential for activating the enzyme prophenoloxidase (PPO) in the melanization response during septic infections. The activation of cSPHs requires their proteolytic cleavage, yet factors that control their activation and the complexity of their interactions within these cascades remain unclear. Here, we report the identification of CLIPA28 as a novel immune-related cSPH in the malaria vector *Anopheles gambiae*. Functional genetic analysis using RNA interference (RNAi) revealed that CLIPA28 is essential for the melanization of *Plasmodium berghei* parasites in refractory mosquitoes, and for mosquito resistance to fungal infections. We further show, using combined biochemical and genetic approaches, that CLIPA28 is member of a network of at least four cSPHs, whereby members are activated in a hierarchical manner following septic infections. Depletion of the complement-like protein TEP1 abolished the activation of this network after septic infections, whereas, depletion of the serine protease inhibitor 2 (SRPN2) triggered enhanced network activation, even in naïve mosquitoes, culminating in a dramatic reduction in cSPHs hemolymph levels, which paralleled that of PPO. Our data suggest that cSPHs are engaged in complex and multilayered interactions within serine protease cascades that regulate melanization, and identify TEP1 and SRPN2 as two master regulators of the cSPH network.

## Author summary

Melanization is a spectacular immune response of insects that culminates in the deposition of melanin on microbial surfaces leading to their death. Despite more than half a century of investigation of this immune reaction in several insect models, important knowledge gaps, specifically concerning the regulation of this response, remain, especially that its output is tightly regulated by complex genetic and biochemical interactions between members of structurally diverse immune gene families. Here, we identify a new hemolymph protein that is involved in the melanization of malaria parasites and show that it forms, together with other proteins of the same gene family, a network, whereby members are activated in an orderly manner during mosquito septic infections. Furthemore, we show that this network is controlled by two structurally distinct immune proteins which act as master regulators of its activation. Dissecting the complexity of the regulatory mechanisms mediating insect melanization responses, may facilitate fine manipulation of mosquito immunity to reduce vectorial capacity.

## Introduction

In insects, key humoral immune responses such as antimicrobial peptide synthesis by the Toll pathway [1], melanization [2–4], and complement-mediated attack [5] are regulated by serine protease cascades composed mainly of clip-domain serine proteases (cSP) that are present in the hemolymph as zymogens. Not all cSPs are catalytic, those that lack one or more of the three residues (His, Asp, Ser) that form the catalytic triad are non-catalytic [also known as clip-domain containing serine protease homologs (cSPHs)]. These cascades have been mostly studied in the context of melanization, an immune effector response that is triggered locally in response to cuticle injury, or systemically following microbial invasion of the hemocoel [6,7]. It is characterized by the synthesis of melanin and its cross-linking with molecules on microbial surfaces, or in injured areas resulting in the killing of the invader, and hardening of the wound clot. A key enzyme in this response is phenoloxidase (PO), which catalyzes the oxidation of tyrosine to dihydroxyphenylalanine, and the oxidation of dihydroxyphenylalanine and dopamine to their respective quinones that act as precursors of melanin formation [reviewed in [8]]. Systemic infections rapidly activate the melanization response by triggering the proteolytic processing of cSPs and cSPHs, leading to prophenoloxidase (PPO) cleavage into active PO by a terminal cSP in the cascade, known as a prophenoloxidase activating proteinase (PAP).

Phylogenetic analysis of clip domain serine proteases in the malaria vector *A*. *gambiae* lead to their classification into 5 groups (A to E); group A includes only cSPHs, groups B, C and D are mainly cSPs, while group E genes are either cSPHs or mixed cSP-cSPHs (i.e. containing both catalytic and non-catalytic domains) [9,10]. Despite being non-catalytic, cSPHs were shown initially to play critical roles in the insect melanization response by mediating the proper activation cleavage of PPO. In *Manduca sexta*, two cSPHs, SPH1 and SPH2, act as cofactors for PAP1 and PAP3 to efficiently cleave and activate PPO [11–13]. The precursor forms of SPH1 and SPH2 cannot activate PPO [13,14], but rather require processing by PAP3 and PAP1 to become active [12,15]. Similarly, *Tenebrio molitor* cSPH1 [16] and *Holotrichia diomphalia* PPAFII [17] are two cSPHs that were also shown to be indispensable for PPO activation. Structural studies revealed that PPAFII interacts directly with PPO forming large molecular weight oligomers, and that this interaction is likely to induce a conformational change in PPO facilitating its cleavage by the cSP PPAF-1 [18]. Subsequent studies in *A*. *gambiae* revealed novel functions for cSPHs that extend beyond PPO activation to regulate the activity of the complement-like protein TEP1 [19,20]. TEP1, a hallmark of mosquito immunity, is responsible for the elimination of the majority of *Plasmodium* ookinete stages through lysis or melanization, depending on the host genetic background [21–23]. Several factors are associated with the regulation of TEP1 activity including, clip-domain serine protease homologs (cSPHs) [20,23], nitric oxide generated by invaded midgut epithelial cells [24], and the release of hemocyte-derived microvesicles [25]. SPCLIP1 [19] (also called CLIPA30 [9]) and CLIPA2 [20] are the two cSPHs that regulate ookinete killing by TEP1 in a positive and negative manner, respectively, through yet unknown mechanisms. SPCLIP1 and CLIPA2 hemolymph protein levels are tightly correlated with the hemolymph activity of TEP1_cut_, the mature processed form of TEP1. A marked reduction in TEP1_cut_ protein observed in *LRIM1* knockdown (kd) naïve mosquitoes [26,27] is paralleled by a similar reduction in SPCLIP1 [19] and CLIPA2 [20]. Results generated from all these studies revealed the existence of significant functional interactions between mosquito complement and clip cascades. On the other hand, all mosquito cSPHs identified so far play key roles in the melanization response, whereby CLIPA2 [20] and CLIPA14 [22] function as negative regulators, while SPCLIP1 [19] and CLIPA8 [28] as positive regulators. Similarly, several cSPs of the CLIPB subfamily were shown to contribute to the melanization response to different extents [29–32]. The substantial number of cSPHs and cSPs involved in melanization suggest that the protease network regulating this response is governed by a staggering complexity of interactions, especially that both cSPHs and cSPs form expanded gene families [9,10]. Here, we identify CLIPA28 as a novel cSPH essential for the mosquito melanization response. CLIPA28 kd significantly reduced *Plasmodium berghei* ookinete melanization in refractory *A*. *gambiae* mosquitoes, in which the *P*. *berghei* agonist gene CTL4 [33] has been silenced. Importantly, we show that CLIPA28 is part of a complex cSPH network in which members undergo activation cleavage in a hierarchical manner. This activation profile in addition to the abundant number of cSPHs in this module, suggest that mosquito cSPHs are likely to interact with cSPs at different levels within the protease network that regulates melanization. We further show that TEP1 and SRPN2, a key negative regulator of the mosquito melanization response, act as master regulators of mosquito cSPHs. Our data provide novel insights into the regulation of CLIP protease cascades in the mosquito hemolymph, which should help decipher the dynamic interactions between the cSP and cSPH components of these cascades.

## Results and discussion

### CLIPA28 is a novel positive regulator of *P*. *berghei* ookinete melanization

**Fig 1:**
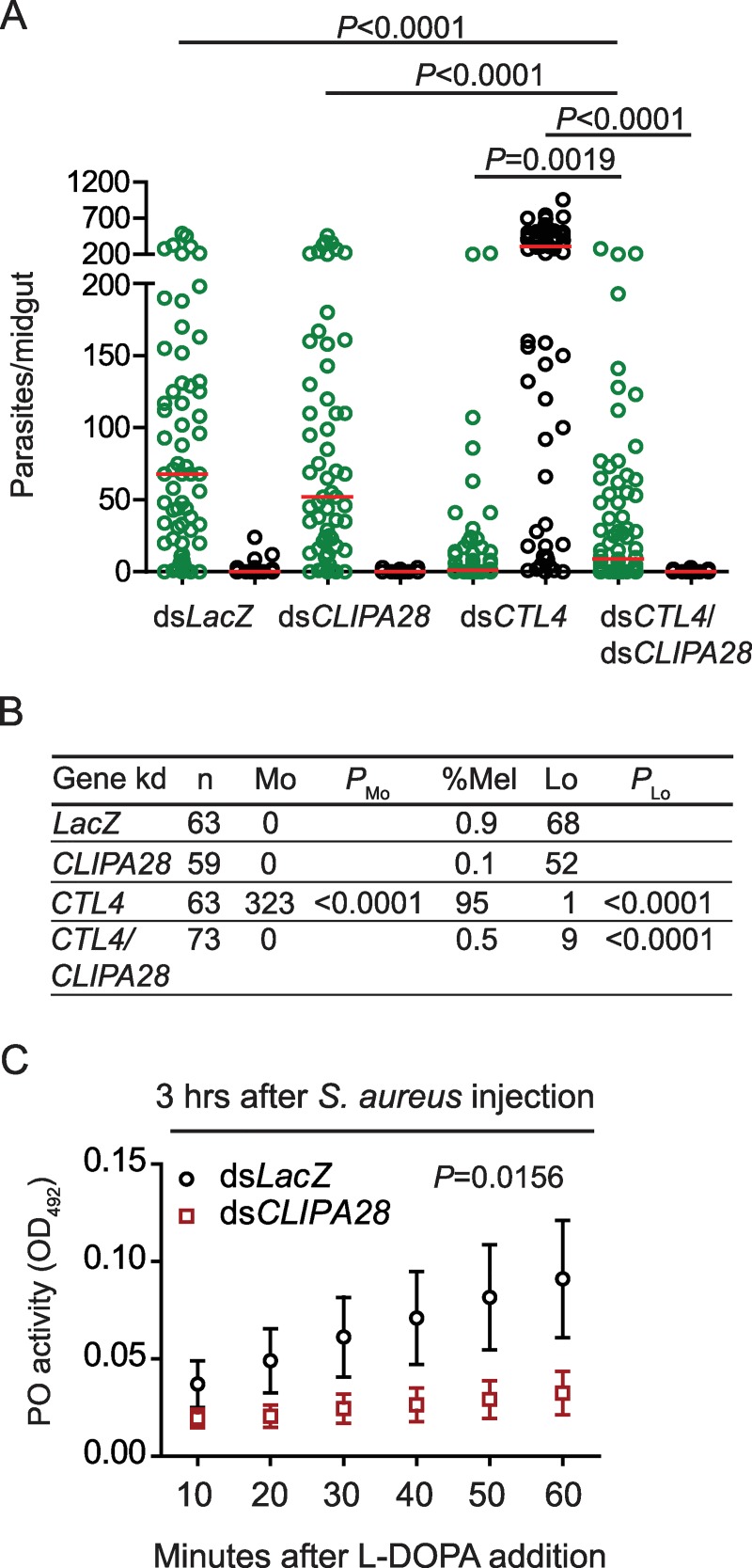
CLIPA28 is required for the melanization of *P*. *berghei* ookinetes. (A) Scatter plots of live GFP-expressing *P*. *berghei* oocysts (green circles) and dead melanized ookinetes (black circles) scored in the midguts of the indicated mosquito genotypes at seven days post-infection. Red lines indicate median parasite numbers. Statistical analysis for the parasite distribution was performed using the Mann-Whitney test, and *P*-values less than 0.05 were considered significant. Data were pooled from four independent biological experiments. (B) Tabulated data of Fig 1A showing the percentage of melanized ookinetes (% Mel), and the median numbers of melanized ookinetes (Mo) and live oocysts (Lo) per midgut. *P* values were calculated using the Mann-Whitney test. (C) Phenoloxidase (PO) enzymatic activity [detected as absorbance at OD_492_, after conversion of L-3,4-dihydroxyphenylalanine (L-DOPA)] was measured in hemolymph extracted from ds*LacZ* (control) and ds*CLIPA28* mosquitoes at 3 hrs post-injection of live *S*. *aureus* (OD_600_ = 0.8). The graph shows PO activity measured at 10 min intervals up to 60 min after addition of L-DOPA. Each point on the graph represents the mean calculated from seven independent biological experiments. Error bars represent standard error of the mean. In each experiment, a separate regression line was fitted for ds*CLIPA28* and ds*LacZ* control. The slopes were compared between both dsRNA treatments using the Wilcoxon signed rank test and medians were considered to be significantly different if *P* < 0.05.

Functional studies in wildtype and refractory *A*. *gambiae* genetic backgrounds allowed the identification of several cSPHs that regulate the melanization of *P*. *berghei* ookinetes, as they egress from midgut epithelial cells into the basal labyrinth. CLIPA8 [30] and SPCLIP1 [19] act as positive regulators, while CLIPA2 [20,30] and CLIPA14 [22] act as negative regulators. To identify novel cSPHs involved in the mosquito melanization response we searched for genes that are significantly co-regulated with *CLIPA2* in a developmental transcriptome data set of Expressed Sequence Tags [34]. Pearson correlation coefficient identified two CLIP clusters with similarity to CLIPA2 developmental expression greater than 0.85: CLIPC7 (PCC 0.867) and AGAP010730 (PCC 0.871). Interestingly, AGAP010730 is a cSPH that is just adjacent to CLIPA8 on chromosome 3L, and shares the highest sequence homology with CLIPA8 and CLIPA9. As we were approaching completion of this work, the gene models of all *A*. *gambiae* serine proteases were improved and AGAP010730 was annotated as CLIPA28 [9]. Hence, we utilized the same gene name for simplicity. To determine whether CLIPA28 is involved in the mosquito melanization response, we asked first whether its kd modulates *P*. *berghei* ookinete melanization in susceptible and *CTL4* kd refractory mosquitoes [33,35]. To that purpose, mosquitoes injected with ds*CLIPA28* (double-stranded RNA specific to CLIPA28), ds*CTL4* and ds*CTL4*/ds*CLIPA28* mixture were infected with *P*. *berghei*, and their midguts dissected at day 7 post-infection to score the numbers of live oocysts and melanized ookinetes. Mosquitoes treated with ds*LacZ* (dsRNA specific to the β-galactosidase gene) were used as control. Silencing *CLIPA28* in a wildtype background did not affect neither the number of live oocysts nor melanized ookinetes relative to control, but it abolished melanization in ds*CTL4* mosquitoes, indicating that CLIPA28 is a key player in the melanization response (Fig 1A and 1B; S1 Table). The fact that ookinete melanization requires also two other cSPHs, SPCLIP1 [19] and CLIPA8 [30], indicates that cSPHs that positively regulate *A*. *gambiae* melanization exhibit unique non-redundant roles. Here, it is worth noting that *P*. *berghei* is a tractable rodent model malaria parasite due to the ease of conducting mosquito infections, the relatively high oocyst load that develops in the midgut, and the susceptibility of this parasite to the melanization response. However, it would also be interesting to compare these results with infections using the human malaria parasite, *P*. *falciparum* for several reasons: First, *A*. *gambiae* immune responses can be parasite species-specific [36]. Second, mosquito immune responses to malaria parasites are infection intensity dependent [35,37], a point particularly pertinent to *P*. *falciparum* that establishes naturally low infection intensities in mosquitoes [38]. CLIPA28, is also required for the activation of hemolymph PPO after bacterial systemic infections; its kd significantly reduced hemolymph PO activity after *S*. *aureus* infections (Fig 1C). We have previously shown that PPO activation in response to bacterial and fungal infections requires CLIPA8 [28,39]. Altogether, these results suggest that CLIPA8 and CLIPA28 play key non-redundant roles in PPO activation in response to diverse microbial challenges, and hence may constitute a core module in the mosquito melanization response.

### CLIPA28 is required for defense against fungal infections

**Fig 2:**
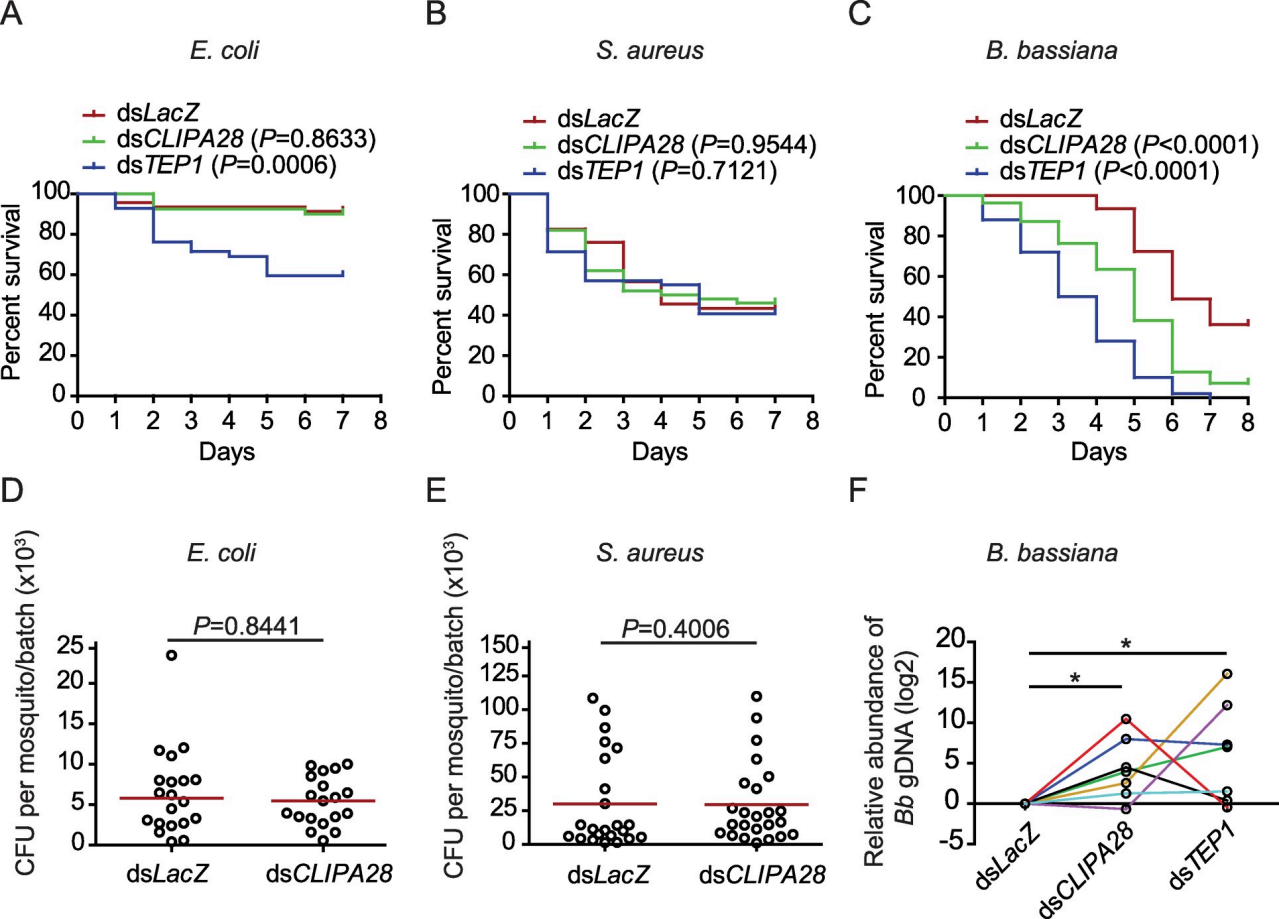
*CLIPA28* kd mosquitoes are susceptible to fungal but not bacterial infections. (A-C) Survival assays of the indicated mosquito genotypes following injection with (A) *E*. *coli* (OD_600nm_ = 0.4),d (B) *S*. *aureus* (OD_600nm_ = 0.4), and (C) after spraying with a *B*. *bassiana* suspension of 1x10^8^ spores/ml. One representative experiment is shown from three independent biological experiments. The Kaplan-Meier survival test was used to calculate the percent survival. Statistical significance of the observed differences was calculated using the Log-rank test. (D-E) Bacterial proliferation assays conducted on mosquitoes injected with (D) *E*. *coli* (OD_600nm_ = 0.8), and (E) *S*. *aureus* (OD_600nm_ = 0.4). Batches of 8 whole mosquitoes each were grinded in LB medium at 48 hrs after infection, and colony forming units (CFU) were scored on LB plates supplemented with the appropriate antibiotic. Each point on the scatter plot represents the mean CFU per mosquito per batch. Statistical analysis was performed using the Mann-Whitney test. Medians (red lines) were considered significantly different if *P* < 0.05. Data shown are from four independent biological experiments. (F) Relative abundance of *B*. *bassiana* genomic DNA measured by real-time PCR in the indicated mosquito genotypes at day 4 after spore injection. Each point on the graph represents the mean relative abundance of *B*. *bassiana* genomic DNA in total DNA extracted from a batch of 8 mosquitoes. Seven different biological experiments are shown, each in a different color. Statistical significance of the observed differences was calculated using the Wilcoxon Signed Rank Test. Asterisk (*) denotes *P*<0.05.

Clip protease cascades regulate multiple immune responses which include, in addition to melanization, coagulation [reviewed in [40]] and Toll pathway activation [reviewed in [1]]. Since certain insect cSPs activate both the PPO and Toll pathways [41–43], investigating the potential anti-microbial functions of cSPHs remains legitimate, as their regulatory roles within clip cascades might influence several immune responses, not only melanization. Silencing *CLIPA28* did not influence neither mosquito tolerance nor resistance to *E*. *coli* (Fig 2A and 2D; S1A Fig), or *S*. *aureus* (Fig 2B and 2E; S1B Fig) infections, a result similar to that obtained previously with CLIPA8 [28]. On the other hand, *CLIPA28* kd rendered mosquitoes more susceptible to infections with the entomopathogenic fungus *Beauveria bassiana*, though to a lesser extent than that of *TEP1*, used herein as positive control (Fig 2C; S1C Fig). Relative quantification of *B*. *bassiana* genomic DNA by real-time PCR revealed that fungal proliferation was significantly enhanced in *CLIPA28* and *TEP1* kd mosquitoes (Fig 2F), which confirms our previous observation that the melanization and complement responses contribute significantly to anti-fungal immunity [39]. Nevertheless, we cannot exclude that the susceptibility to fungal infections in *CLIPA28* kd mosquitoes may also be due to regulation of cSP cascades acting upstream of the mosquito Toll/Rel1 pathway, especially that the role of Rel1 in anti-fungal immunity has been established in *A*. *gambiae* [44] and *Ae*. *aegypti* [45]. However, at present, it is difficult to address this point, because our knowledge of Toll signaling pathway in *A*. *gambiae* is too fragmentary, and specific gene expression signature-readouts for pathway activation remain absent.

### A positive regulatory cSPH module exhibits a hierarchical mode of activation

**Fig 3:**
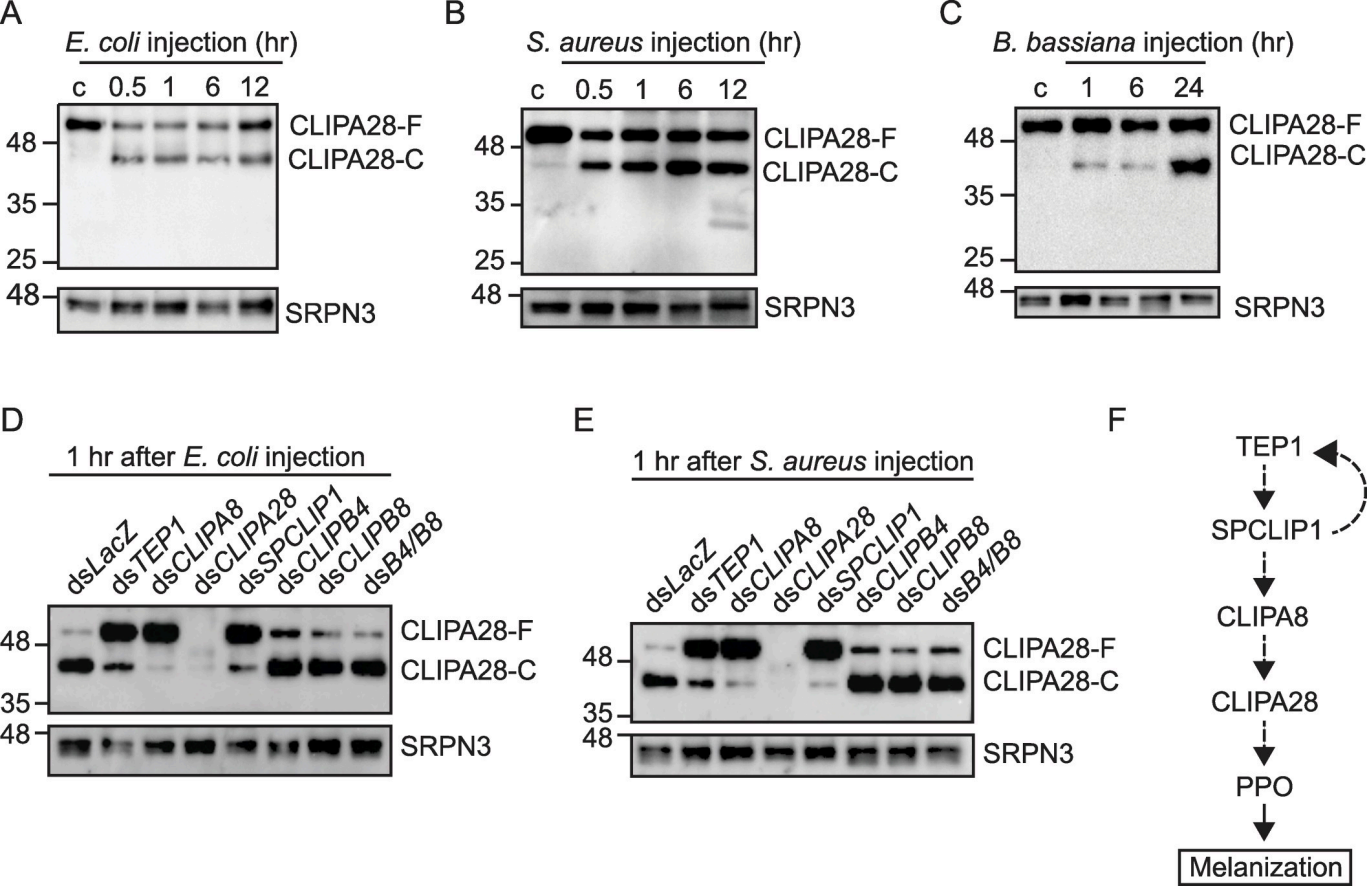
CLIPA28 infection-induced cleavage is controlled by TEP1 and key immune cSPHs. (A-C) Western blots showing CLIPA28 cleavage in wildtype mosquitoes at the indicated time points after injection with (A) *E*. *coli* (OD_600_ = 0.8), (B) *S*. *aureus* (OD_600_ = 0.8) and (C) *B*. *bassiana* (2000 spores/mosquito). (D and E) Western blots showing CLIPA28 cleavage in the indicated mosquito genotypes at 1 hr after injection with (D) *E*. *coli* (OD_600_ = 0.8) and (E) *S*. *aureus* (OD_600_ = 0.8). In all blots, each lane contained hemolymph extracts from 25 mosquitoes. Membranes were stripped and reprobed with αSRPN3 as loading control. (F) Schematic diagram of the hierarchical activation of the positive regulatory cSPHs. Dashed lines indicate that the enzymatic steps are not yet fully characterized.

Biochemical analysis in several insect species including *Manduca sexta* [13–15] and *Tenebrio molitor* [16] revealed that cSPHs require cleavage between the clip and protease domains to become functional, despite being non-catalytic. Here, we show that CLIPA28 is also rapidly cleaved following septic infections with *E*. *coli* (Fig 3A; S2A Fig), *S*. *aureus* (Fig 3B; S2B Fig) and *B*. *bassiana* (Fig 3C; S2C Fig). In the latter, the pronounced cleavage observed at 24 hr coincides with the time at which mycelial growth becomes prominent as previously described [39]. It was noted that at later time points after *S*. *aureus* and *B*. *bassiana* infections, CLIPA28 cleavage coincided with a substantial reduction in hemolymph PPO levels (S2B and S2C Fig), suggesting that these two classes of microbes trigger a more potent melanization response in the host than *E*. *coli*.

To identify candidate genes, specifically cSPs, that may be required for CLIPA28 cleavage, we co-immunoprecipitated (co-IP) CLIPA28 from the hemolymph of mosquitoes sprayed with *B*. *bassiana* spores (i.e. mimicking a natural route of infection), and identified the co-IPed proteins by mass spectrometry. Several immunity proteins with known functions in melanization were identified (S2 Table) including, CLIPB4 [30], CLIPB8 [31,32], TEP1 [21], CLIPA2 [20,30], CLIPA14 [22], and SRPN2 [46]. First, we asked whether the positive regulators CLIPB4, CLIPB8 and TEP1 are required for CLIPA28 cleavage following septic infections with *E*. *coli* and *S*. *aureus*. We also included in this analysis SPCLIP1 and CLIPA8 which despite being absent from the list of proteins that co-IPed with CLIPA28, are key positive regulators of melanization [19,28]. CLIPA28 cleavage after septic infections was not affected neither by the single nor the double kds of *CLIPB8* and *CLIPB4* (Fig 3D and 3E), although both genes were efficiently silenced by RNAi (S3D and S3E Fig). On the other hand, the kds of *CLIPA8*, *TEP1* or *SPCLIP1* strongly inhibited CLIPA28 cleavage (Fig 3D and 3E). We have previously shown that CLIPA8 is similarly cleaved following septic infection [28]. Here, we show that this cleavage pattern is strongly dependent on both *SPCLIP1* and TEP1, as previously reported [19], but not on CLIPA28 (S4A and S4B Fig). Altogether, these data indicate that the positive regulatory cSPHs are activated in a hierarchical manner, with SPCLIP1 being upstream followed by CLIPA8 then CLIPA28 (Fig 3F). These data and those from previous studies [19,20,39] strongly support the most upstream position, so far, for TEP1 in the melanization response. The upstream position of SPCLIP1 among the positive regulatory cSPHs was not surprising since it was previously shown to regulate TEP1 accumulation on microbial surfaces, despite being downstream of TEP1 [19]. Three other cSPs appeared in the list of CLIPA28 co-IPed proteins; CLIPD1, CLIPB13 and CLIPB5. The latter was not included in the analysis since previous functional genetic analysis did not reveal any role for this gene in parasite melanization [30], whereas neither *CLIPB13* (S5A and S5B Fig) nor *CLIPD1* kd (S5C Fig) affected CLIPA28 cleavage. We tested three other cSPs, CLIPB9, CLIPB14, and CLIPB17, which did not appear in the list of CLIPA28 co-IPed proteins, but are known to play key roles either in parasite melanization (CLIPB14 [31] and CLIPB17 [30]) or tissue melanization induced by *SRPN2* kd (CLIPB9 [29]). Yet, none of them was required for CLIPA28 cleavage (S5A and S5B Fig). It is intriguing that none of the cSPs known to play key roles in melanization is required for CLIPA28 cleavage. These results suggest that cSPs may exhibit functional redundancy with respect to cSPH cleavage, or certain cSPHs may act upstream of cSPs to regulate their activation. Although there is no literature support for either possibility, mainly because the cSPH gene family has not been subjected to rigorous genetic and biochemical analysis in other insect species, as it has in *A*. *gambiae*; nevertheless, the clear pattern of hierarchical activation of mosquito cSPHs suggests that a multilayered interaction with cSPs to control the activation of downstream effectors, such as PPO, is not unexpected. We cannot exclude that the absence of control over CLIPA28 cleavage by candidate cSPs may be due to the incomplete silencing of these genes (S3E Fig), which could not be verified at the protein level due to the lack of antibodies for most of them, except for CLIPB8. However, the fact that, at least, CLIPB17 [30], CLIPB14 [31] and CLIPB9 [29] have clear RNAi phenotypes *in vivo*, suggests that this possibility remains minor.

### The positive regulatory cSPH module and CLIPA2 regulate CLIPA14 cleavage

**Fig 4:**
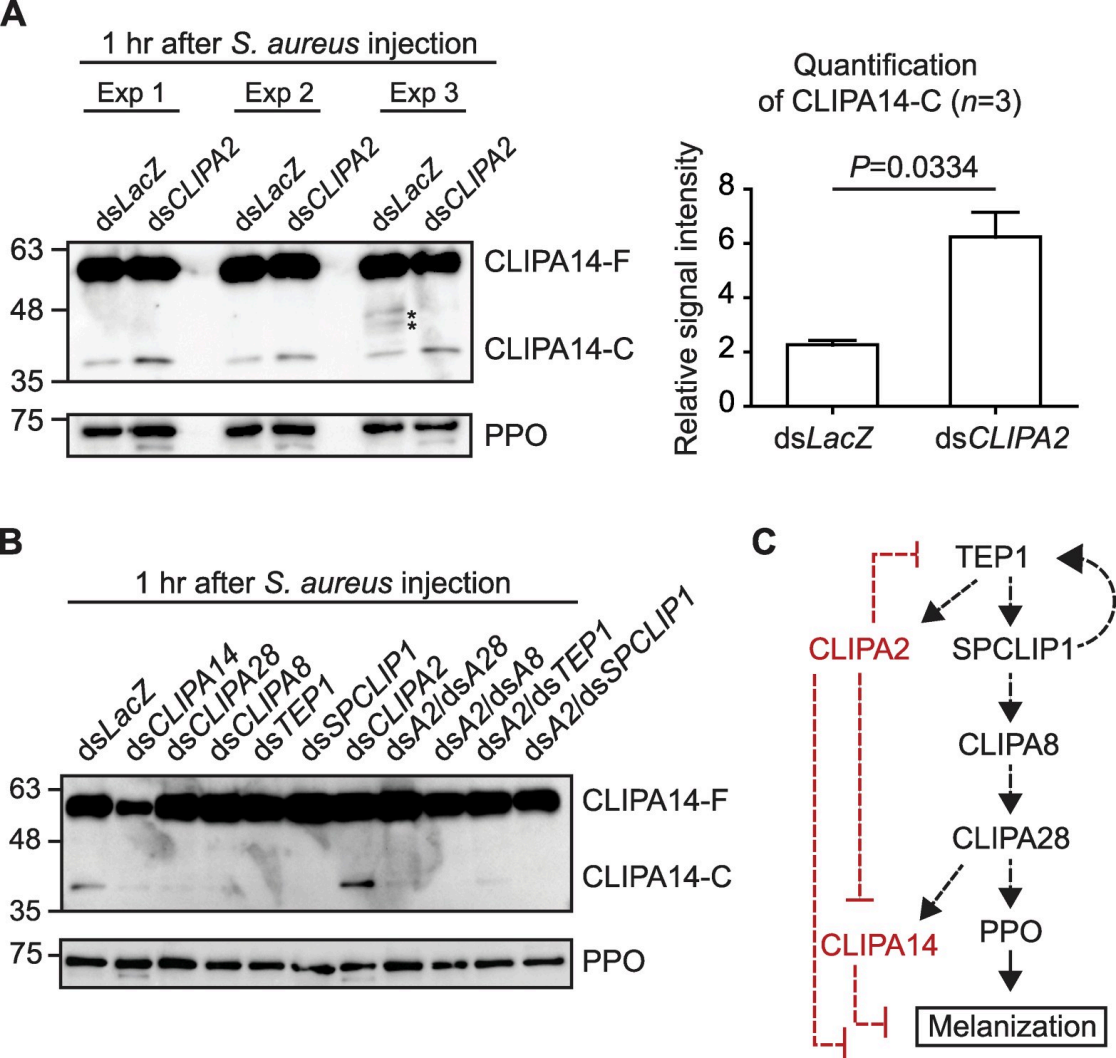
CLIPA14 is downstream of the mosquito cSPHs involved in melanization. (A) Western blot showing CLIPA14 cleavage in *dsLacZ* and *dsCLIPA2* mosquitoes in three different experiments. Protein quantification was performed using the Bradford protein assay and equal amounts of hemolymph proteins were loaded per lane. The graph represents the quantification of CLIPA14-C from these three experiments using Image Lab 5.0. The Y-axis represents the relative signal intensity. Statistical analysis was performed using the Student's paired t-test and differences were considered to be significant if *P* < 0.05. Asterisks (*) indicate non-specific bands. (B) Representative western blot showing CLIPA14 cleavage in the hemolymph of the indicated mosquito genotypes at 1 hr after *S*. *aureus* (OD_600_ = 0.8) injection. Each lane contained hemolymph extracts from 25 mosquitoes. αPPO6 antibody was used to control for loading. The image is representative of two independent biological experiments. (C) Schematic diagram of the hierarchical activation of the positive and negative (in red) regulatory cSPHs. Dashed lines indicate that the enzymatic steps are not yet fully characterized.

CLIPA14 is a negative regulator of melanization in *A*. *gambiae*, and is cleaved following septic infections in a TEP1 and SPCLIP1-dependent manner [22]. Here, we show that CLIPA14 cleavage is negatively regulated by CLIPA2 whose knockdown enhanced CLIPA14 cleavage in response to *S*. *aureus* infections by approximately 2.7 folds (Fig 4A). On the other hand, CLIPA14 cleavage is positively regulated by TEP1, SPCLIP1, CLIPA8 and CLIPA28 in wildtype and *CLIPA2* kd mosquitoes (Fig 4B; S6 Fig), indicating that CLIPA14 is downstream of the positive regulatory cSPHs (Fig 4C). Hence, it seems that CLIPA14 is subject to multiple regulations by positive and negative regulatory cSPHs (Fig 4C), possibly reflecting the key role it plays in the melanization response as inferred from its RNAi phenotype, which is characterized by potent ookinete melanization and exaggerated hemolymph PO activity following septic infections [22]. The placement of CLIPA14, a negative regulator, under the control of positive regulatory cSPHs is possibly a mean to fine tune the intensity of the melanization response in a way to minimize fitness costs on the host. Indeed, a deregulated melanization response due to silencing specific serpins exhibited significant fitness costs in *A*. *gambiae* [46] and *Drosophila* [47,48], manifested by spontaneous tissue melanization and compromised survival. Along the same lines, *CLIPA2* kd mosquitoes exhibited infection-induced reduction in fecundity [20]. Despite the key roles of SPCLIP1 and CLIPA2 in the melanization and complement responses [19,20], their exact placement within the cSPH module could not be established because the antibodies in hand do not recognize the reduced forms of these proteins, and hence, cannot be used to characterize their cleavage profiles. However, since SPCLIP1 and CLIPA2 act as positive and negative regulators of TEP1 accumulation on microbial surfaces, respectively [19,20], and the fact that both cSPHs are almost depleted from the hemolymph of naïve *LRIM1* kd mosquitoes concomitant with the loss of TEP1_cut_ [19,20], supports their upstream position within the cSPH module (Fig 4C).

### SRPN2 is a master negative regulator of mosquito cSPHs

**Fig 5:**
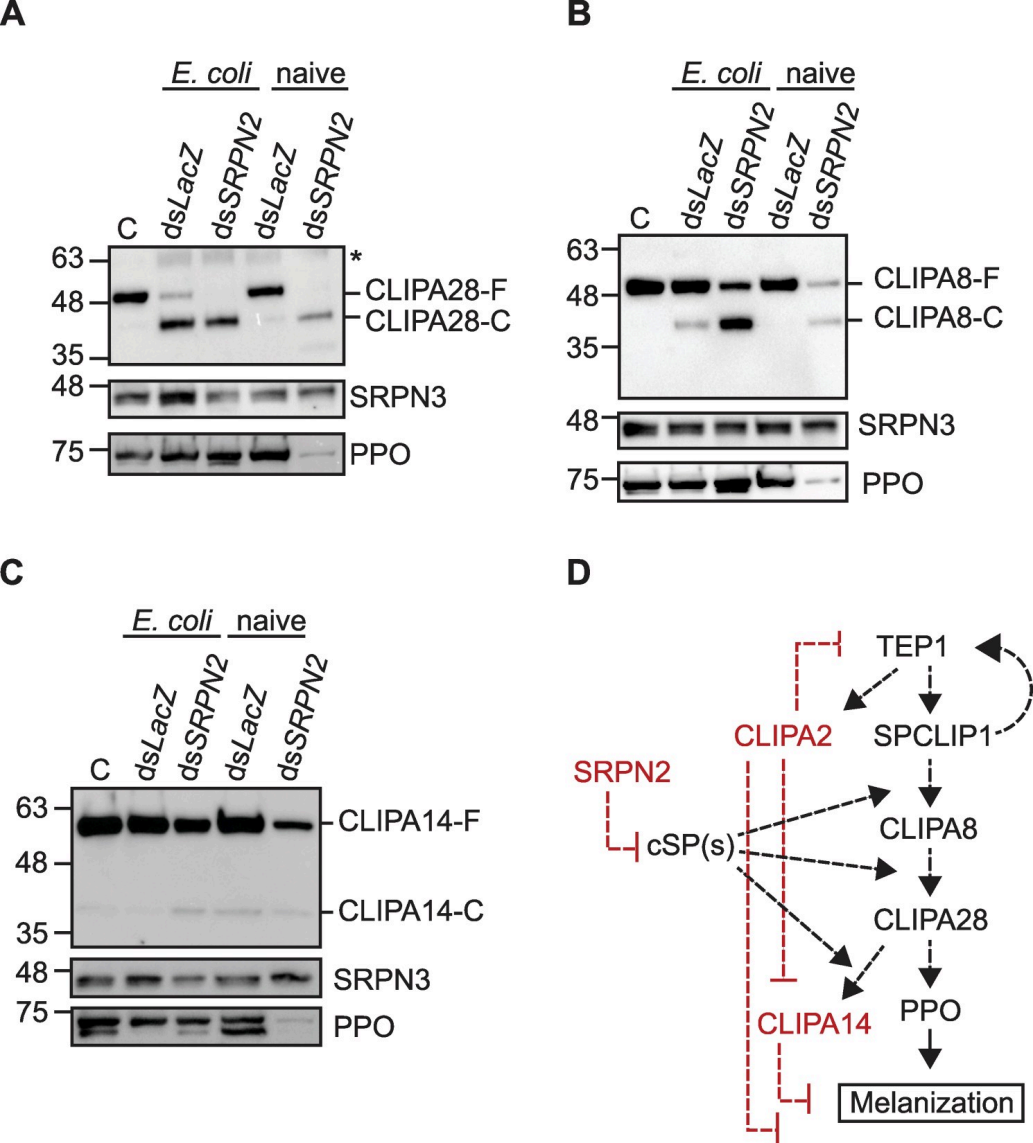
SRPN2 is a negative regulator of cSPH activation cleavage. (A-C) Western blots showing full-length and cleaved forms of (A) CLIPA28, (B) CLIPA8, and (C) CLIPA14 after 1 hr of *E*. *coli* injection in mosquitoes treated for 4 days with *dsLacZ* or *dsSRPN2*, and in naïve mosquitoes at day 7 after treatment with *dsLacZ* or *dsSRPN2*. In each experiment, equal amounts of hemolymph proteins were loaded per lane. Membranes were also probed with αPPO6 to monitor PPO dynamics in *SRPN2* kd mosquitoes, and αSRPN3 for loading control. (D) Schematic diagram showing the putative position of SRPN2 as a negative regulator of the yet unidentified cSP(s) involved in the proteolytic processing of CLIPA8, CLIPA28 and CLIPA14. Factors with negative regulatory roles in melanization are shown in red. Dashed lines indicate that the enzymatic steps are not yet fully characterized. *C*, naïve wildtype-mosquitoes. Asterisk (*) indicates non-specific bands.

SRPN2 is a key negative regulator of the mosquito melanization response; its knockdown elicits a strong melanotic response against *Plasmodium* ookinetes, and substantial tissue melanization in naïve mosquitoes, with the concomitant depletion of PPO from the hemolymph, especially at later time points [46]. To determine whether SRPN2 regulates the cleavage of mosquito cSPHs, we silenced SRPN2 in female *A*. *gambiae* mosquitoes and monitored the cleavage profiles of CLIPA8, CLIPA28 and CLIPA14 under septic and naïve conditions. For septic infections, mosquitoes were challenged with *E*. *coli* at day 4 after treatment with ds*SRPN2* or ds*LacZ* (control). At 1 hr after *E*. *coli* challenge, *SRPN2* kd had a modest effect on the cleavage of CLIPA28 and CLIPA14 (Figs 5A, 5C, S7A and S7C), as manifested by a slight reduction in their full-length forms. However, *SRPN2* kd strongly increased the cleavage of CLIPA8, as shown from the enrichment of the cleaved CLIPA8-C form (Figs 5B and S7B). Interestingly, the full-length forms of these cSPHs were dramatically reduced in naïve mosquitoes at day seven after *SRPN2* kd (Fig 5A–5C; S7A–S7C Fig) relative to control, suggesting an enhanced consumption by the exaggerated melanization response known to be elicited in this mosquito genotype, especially at late time points (beyond day 4) [46]. Despite this enhanced cSPH activation in naïve *SRPN2* kd mosquitoes, we did not observe a substantial enrichment of their cleaved forms, probably because of their sequestration in the melanotic bodies that develop in various tissues of *SRPN2* kd mosquitoes [46]. This enhanced consumption of cSPHs parallels that of PPO (Fig 5A–5C; S7A–S7C Fig), indicating that SRPN2 is critical to keep these cSPHs inactive in naïve mosquitoes to prevent generalized PPO activation. These results also suggest that SRPN2 is a master regulator of the cSPs involved in the processing of CLIPA28, CLIPA8 and CLIPA14, and hence acts upstream of the mosquito cSPH module that controls melanization (Fig 5D). However, so far, only CLIPB9 was shown to form inhibitory complexes with SRPN2 that are physiologically relevant [29]. Yet, *CLIPB9* kd did not affect the cleavage of CLIPA28 (S5 Fig) suggesting that it might be either acting in a different branch of the melanization response, or significant redundancy exists among cSPs with respect to cSPH cleavage, as discussed in the previous section. The use of biochemical approaches for the identification of target proteases for individual serpins remains challenging in small insects with limited amounts of hemolymph, due to rapid clearance of these complexes from the hemolymph [49,50], whereas such a strategy was met with a greater success in larger insects like *M*. *sexta* [51].

In conclusion, the hierarchical activation of cSPHs informs a multilayered control over the cSP cascade involved in the infection-induced melanization response. cSPs and cSPHs are likely to interact at different levels and their molecular interactions are at the core of the protease network that controls melanization, and possibly other immune responses, including Toll pathway activation and coagulation. The substantial number of mosquito cSPHs involved in melanization and their hierarchical cleavage profile raise the hypothesis that, in addition to their classical role in directly controlling proper PPO cleavage, cSPHs might also confer specificity and order to the cleavage of cSPs, possibly as a mean to counter a random flow of information within these protease networks, due to partial functional redundancies among the cSPs themselves. Evidence for such redundancy is provided by the RNAi phenotypes of certain cSPs that lead to partial reversion of ookinete melanization in refractory *A*. *gambiae* mosquitoes [30]. Functional redundancy among cSPs regulating Toll pathway activation has been recently demonstrated in *Drosophila*, using an elegant genetic approach [52]. An additional layer of complexity in studying these protease networks is that their components, including cSPHs, cSPs and SRPNs, and their putative target PPOs and TEPs belong to large gene families [10]. Nevertheless, analysis of the cleavage profiles and biochemical functions of cSPs and cSPHs are expected to provide a more comprehensive understanding of these networks that would allow fine manipulation of mosquito immunity.

## Materials and methods

### Ethics statement

This study was carried according to the recommendations in the Guide for the Care and Use of Laboratory Animals of the National Institutes of Health (Bethesda, USA). Animal protocol was approved by the Institutional Animal Care and Use committee IACUC of the American University of Beirut (permit number 17-10-451). The IACUC functions in compliance with the Public Health Service Policy on the Humane Care and Use of Laboratory Animals (USA), and adopts the Guide for the Care and Use of Laboratory Animals of the National Institutes of Health.

### *Anopheles gambiae* rearing and *P*. *berghei* infections

All experiments were performed with adult female *Anopheles gambiae* G3 strain mosquitoes. Mosquitoes were maintained at 27 (±1)°C and 75 (±5) % humidity with 12-hour day-night cycle. Larvae were reared in 752 cm^2^ plastic pans at a density of approximately 150 larvae per pan and given tropical fish food. Freshly emerged adult mosquitoes were collected from larval pans using a vacuum collector, maintained on 10% sucrose, and given BALB/c mice blood (mice were anesthetized with ketamine) for egg laying. Mosquito infections with *P*. *berghei* (GFP-expressing strain PbGFPCON [53]) were performed by allowing mosquitoes to feed on 5–6 week old anesthetized BALB/c mice infected with *P*. *berghei* at a parasitemia of 4–6% for approximately 20 min at 20°C. Mosquitoes were then maintained on 10% sucrose solution at 20°C until they were dissected. Mosquito midgut dissections and scoring of live GFP-positive oocysts and dead melanized ookinetes were performed as previously described [30]. Data were collected from four independent biological experiments and statistical significance was calculated using the Mann-Whitney test. Means were considered significantly different if *P*<0.05.

### Gene silencing by RNA interference

Double stranded RNA (dsRNA) synthesis was performed using the T7 RiboMax Express Large Scale RNA production system (Promega) according to the manufacturer’s instructions, and dsRNAs were purified as previously described [21]. Primers used for dsRNA production are listed in S3 Table. *In vivo* gene silencing was performed as previously described [54]. Mosquitoes were microinjected with a 69nl of a 3μg/μl solution of gene-specific dsRNA for single gene kd, or 138 nl of a solution containing a mixture of two dsRNAs (each at a concentration of 3μg/μl) for double gene kds. Efficiency of gene silencing was measured by quantitative Real-time PCR (qRT-PCR), or western blot analysis whenever antibodies are available (S3 Fig). Concerning qRT-PCR, total RNA was isolated using TRIzol reagent from 15 mosquitoes per experimental sample. RNA extraction, first strand cDNA synthesis and qRT-PCR were performed as previously described [55]. Primers used to score the efficiency of gene silencing are listed in S4 Table. Relative gene expression values were calculated using the comparative C_T_ method after checking for the efficiency of target amplification. To measure the efficiency of gene kd at the protein level, hemolymph was extracted from approximately 20 naïve mosquitoes four days after dsRNA injection and analyzed by western blot as described below.

### Survival and microbial proliferation assays

The survival of dsRNA-treated adult female mosquitoes was scored over a period of 8 days after intrathoracic injection of a suspension of ampicillin-resistant *E*. *coli* strain OP-50 (OD_600_ = 0.4) [56], or tetracyclin-resistant *S*. *aureus* strain (OD_600_ = 0.4) [28] in PBS, or after spraying with a suspension of *B*. *bassiana* (strain 80.2) containing 1x10^8^ conidia/ml in 0.05% Tween-80 prepared as previously described [39,57]. The Kaplan-Meier survival test was used to calculate the percent survival. Statistical significance of the observed differences was calculated using the log-rank test. Experiments were repeated at least 3 times using different batches of mosquitoes. For the bacterial proliferation assays, dsRNA-treated mosquitoes were injected intrathoracically with 69nl of ampicillin-resistant *E*. *coli* strain OP-50 (OD_600_ = 0.8) or tetracycline-resistant *S*. *aureus* strain (OD_600_ = 0.4) for 48 hr. At least 4 batches of 8 mosquitoes each per genotype were grinded in 400μl Luria Bertani (LB) Broth at 48 hr after *E*. *coli* and *S*. *aureus* injections. Serial dilutions of mosquito homogenates were plated onto LB agar selection plates containing the appropriate antibiotic, and colony-forming units (CFUs) were scored. Data shown are from four independent biological experiments. Statistical significance was calculated using the Mann-Whitney test. Medians were considered significantly different if *P*<0.05. The proliferation of *B*. *bassiana* in spore-infected mosquitoes was scored by qRT-PCR as follows. Briefly, *dsLacZ*, *dsTEP1* and *dsCLIPA28*-treated mosquitoes were injected each with approximately 30 *B*. *bassiana* spores in PBS. Four days later, whole mosquitoes were grinded in liquid nitrogen with a mortar and pestle to create a fine powder. The powder was collected into Eppendorf tubes and genomic DNA was extracted using the CTAB buffer (0.1M Tris-HCl, pH 8.0, 0.01M EDTA, 1.4M NaCl, 2% cetyltrimethyl ammonium bromide), as previously described [58]. QRT-PCR was performed in a CFX96 Real-Time Detection System (Bio-Rad) using the SYBR Green JumpStart^TM^ Taq ReadyMix (Sigma-Aldrich), according to the manufacturer’s instructions. *B*. *bassiana* primers used in qRT-PCR are the following: Bb_ITSII_F: 5’-GCC GGC CCT GAA ATG G-3’and Bb_ITSII_R: 5’- GAT TCG AGG TCA ACG TTC AGA AG-3 [59]. The *A*. *gambiae* ribosomal S7 gene was used as an internal control for normalization using the primers, AgS7-F: 5’-AGAACCAGCAGACCACCATC-3’and AgS7-R: 5’-GCTGCAAACTTCGGCTATTC-3’ [60]. Relative gene expression values were calculated using the Comparative C_T_ method after checking for the efficiency of target amplification.

### Expression of CLIPA28 in SF9 cells and generation of CLIPA28 antibodies

The entire CLIPA28 open reading frame lacking the endogenous signal peptide and stop codon was amplified from *A*. *gambiae* cDNA using the following primer pair: AGAP010730 LIC-F, 5'-GACGACGACAAGATGCAAGACATTGAAGAAGAACTG-3' and AGAP010730 LIC-R, 5'-GAGGAGAAGCCCGGTTTCAATTTTATATCAAAACTCTC-3'. The underlined sequences are extensions to allow ligase-independent cloning in *pIEx10* insect cell expression plasmid (Novagen) as a fusion with an N-terminal streptavidin-tag and C-terminal His-tag according to the manufacturer’s protocol. Transfection of Sf9 cells with *pIEx10-CLIPA28* and purification of recombinant CLIPA28^HIS^ was performed as previously described [20]. Purified recombinant CLIPA28^HIS^ protein was used to generate rabbit polyclonal antibody (Eurogentec). CLIPA28 antibody was affinity purified over an AminoLink column (Pierce) containing covalently bound CLIPA28^HIS^ according to the manufacturer’s protocol.

### Western blot analysis

Adult female *A*. *gambiae* mosquitoes were injected each with 69 nl of *E*. *coli* (OD_600_ = 0.8) or *S*. *aureus* (OD_600_ = 0.8) suspension in PBS, or with 2000 spores of *B*. *bassiana* suspension in water, prepared as previously described [57]. Hemolymph was generally collected from 25 mosquitoes per sample by proboscis clipping directly into protein loading dye (1x). However, for monitoring the cleavage profiles of CLIPA14, CLIPA28 and CLIPA8 in *SRPN2* kd mosquitoes, and the cleavage profile of CLIPA14 in *CLIPA2* kd mosquitoes, hemolymph was extracted from 25 mosquitoes into ice-chilled PBS containing a cocktail of protease inhibitors (Roche), and protein quantification was performed using the Bradford protein assay (Fermentas) to ensure equal loading per well. Proteins were resolved by SDS-PAGE and transferred to immunoblot PVDF membrane using wet transfer (BioRad). Primary antibodies used for immunoblotting are mouse αCLIPA8 [28], rabbit αCLIPA28, rabbit αCLIPA14 [22], rabbit αPPO6 [61], rabbit αCLIPB8 [32] (kind gift from Kristin Michel), rabbit αSRPN2 [46] and rabbit αSRPN3 [28] added overnight at the following dilutions 1:30, 1:2000, 1:1000, 1:2000, 1:1000, 1:1000 and 1:1000, respectively. A lower band is sometimes observed with αPPO6 which may indicate cleaved form of the zymogen [61]. Following washing, blots were incubated for 1 hr with anti-mouse and anti-rabbit IgG horseradish peroxidase-conjugated secondary antibodies at dilutions of 1:6000 and 1:12000, respectively. Blots were then washed, immersed in BioRad Clarity Max western ECL substrate and imaged using ChemiDoc MP (BioRad). Band quantification was performed using Image Lab software.

### Phenoloxidase enzymatic assay

The phenoloxidase enzymatic assay was performed 3 hr after mosquito injections with *S*. *aureus* (OD_600_ = 0.8), using 5–8 μg of mosquito hemolymph per reaction, as described previously [28]. The absorbance at 492 nm was measured at 10 minute intervals (up to 1 hr) after incubation with L-3,4-dihydroxyphenlalanine(L-DOPA) (Sigma) in a MultisKan Ex microplate reader (Thermo Labsystems). The experiment was repeated seven times using different batches of mosquitoes and *S*. *aureus* cultures.

### Co-immunoprecipitation and mass spectrometry

Co-immunoprecipitation (coIP) reactions were performed exactly as previously described [55], using the Pierce crosslink Magnetic IP/co-IP kit. Briefly, hemolymph was collected from approximately 900 female mosquitoes by proboscis clipping at 48 hrs after mosquito spraying with *B*. *bassiana* conidial suspension (i.e. mimicking natural infection route) in water containing 1x10^8^ conidia/ml. Hemolymph extracts were centrifuged at 4000 g for 5 min to remove mosquito and fungal cells and incubated overnight at 4 ° C with a 1:1 slurry of PBS containing agarose beads cross-linked to 4 μg of purified CLIPA28 antibody. Elution of bound proteins, precipitation with trichloroacetic acid and protein identification by mass spectrometry (IGBMC proteomic platform, Strasbourg) were performed exactly as previously described [55].

## Supporting information

S1 Fig *CLIPA28* kd mosquitoes are susceptible to fungal but not bacterial infections. (A-C) Survival assays of the indicated mosquito genotypes following injection with (A) *E*. *coli* (OD_600nm_ = 0.4), (B) *S*. *aureus* (OD_600nm_ = 0.4), and (C) after spraying with a *B*. *bassiana* suspension of 1x10^8^ spores/ml. Two representative experiment are shown for each treatment. The Kaplan-Meier survival test was used to calculate the percent survival. Statistical significance of the observed differences was calculated using the Log-rank test.
(EPS)

S2 Fig CLIPA28 is rapidly cleaved after septic infections. (A-C) Western blots showing CLIPA28 cleavage at the indicated time points after mosquito injection with *E*. *coli* (OD600 = 0.8), *S*. *aureus* (OD600 = 0.8), and *B*. *bassiana* (2000 spores/mosquito). In all experiments, each lane contained hemolymph extracts from 25 mosquitoes. Membranes were also probed with αPPO to monitor PPO dynamics.
(EPS)

S3 Fig Efficiency of gene silencing by RNAi. (A-D) Representative western blots showing the knockdown efficiencies of CLIPA2, TEP1, SPCLIP1, SRPN2 and CLIPB8 in naïve mosquitoes at day four after dsRNA injection. αSRPN3 or αPPO6 were used to control for loading. (E) Efficiency of silencing of the indicated genes measured by qRT-PCR. Data shown are from at least 3 independent experiments. Error bars represent standard error of the mean.
(EPS)

S4 Fig CLIPA28 is downstream of CLIPA8. Western blots showing CLIPA8 cleavage in the indicated mosquito genotypes at 1 hr after injection with (A) *E*. *coli* (OD_600_ = 0.8), and (B) *S*. *aureus* (OD_600_ = 0.8). In all experiments, each lane contained hemolymph extracts from 25 mosquitoes. Membranes were stripped and reprobed with αSRPN3 as loading control.
(EPS)

S5 Fig CLIPA28 cleavage is not affected by candidate *CLIPB* knockdowns. (A-C) Western blots showing CLIPA28 cleavage in the indicated mosquito genotypes at 1 hr after *E*. *coli* (OD_600_ = 0.8) injection. (C) Two independent biological experiments are shown performed with different mosquito batches. In all the western blots, each lane contained hemolymph extracts from 25 mosquitoes. αPPO6 was used to control for loading.
(EPS)

S6 Fig The positive regulatory cSPHs and CLIPA2 regulate CLIPA14 cleavage. A representative western blot showing CLIPA14 cleavage in the hemolymph of the indicated gene kd mosquitoes at 1 hr after *S*. *aureus* (OD_600_ = 0.8) injection. Each lane contained hemolymph extracts from 25 mosquitoes. αPPO6 was used to control for loading.
(EPS)

S7 Fig SRPN2 is a negative regulator of cSPH activation cleavage. Western blots showing full-length and cleaved forms of (A) CLIPA28, (B) CLIPA8, and (C) CLIPA14 in *dsLacZ* or *dsSRPN2* mosquitoes at 1 hr after *E*. *coli* injection, and in naïve mosquitoes at day 7 after treatment with *dsLacZ* or *dsSRPN2*. In each experiment, equal amounts of hemolymph proteins were loaded per lane. Membranes were also probed with αPPO6 to monitor PPO dynamics in *SRPN2* kd mosquitoes.
(EPS)

S1 Table Counts of live oocysts and melanized ookinetes in ds*LacZ* (control), ds*CLIPA28*, ds*CTL4* and ds*CTL4*/ds*CLIPA28* mosquitoes. Raw data are shown from four independent biological expeirments.
(XLSX)

S2 Table List of proteins that co-immunoprecipitate with CLIPA28. (XLS)

S3 Table Primers used for dsRNA production. (DOCX)

S4 Table Primers used in real-time PCR. (DOCX)
