# Supplementary material for: The mosquito melanization response requires hierarchical activation of non-catalytic clip domain serine protease homologs
Source: PLoS Pathog. 2019 Nov 25;15(11):e1008194. doi: 10.1371/journal.ppat.1008194 (PMC6901238; doi:10.1371/journal.ppat.1008194)
Supplement: S3 Table — (DOCX) [file ppat.1008194.s010.docx]

| **S2 Table: Primers used for dsRNA production** | | |
| --- | --- | --- |
| Gene | Primers used for dsRNA synthesis (T7 promoter sequence underlined) | Reference |
| *LacZ* | For: 5'-TAATACGACTCACTATAGGGAGAATCCGACGGGTTGTTACT-3'  Rev: 5'-TAATACGACTCACTATAGGGCACCACGCTCATCGATAATTT-3' | [[1](#_ENREF_1)] |
| *TEP1*  (AGAP010815) | For: 5'-TAATACGACTCACTATAGGGTTTGTGGGCCTTAAAGCGCTG-3'  Rev: 5'- TAATACGACTCACTATAGGGACCACGTAACCGCTCGGTAAG-3' | [[2](#_ENREF_2)] |
| *CTL4*  (AGAP005335) | For: 5'- TAATACGACTCACTATAGGGGTTAGCAGCATTGGGATTACCCT-3'  Rev: 5'- TAATACGACTCACTATAGGGGAAGTCGCAACCCAGCTCATTGT-3' |  |
| *SPCLIP1*  (AGAP028725) | For: 5'- TAATACGACTCACTATAGGGGTCACCGAACACGGCCAAC-3'  Rev:5'-TAATACGACTCACTATAGGGATCGAAGCTGATCGGATCGGG-3' | [[3](#_ENREF_3)] |
| *CLIPA2*  (AGAP011790) | For:5'- TAATACGACTCACTATAGGGATCCTAACAACGGCACACTGTGTGA-3'  Rev:5'-TAATACGACTCACTATAGGGTCCTGATCGCCATGATTGGTGGTGCT-3' | [[4](#_ENREF_4)] |
| *CLIPA14*  (AGAP011788) | For: 5'- TAATACGACTCACTATAGGGCGGCATCATCGACATCCGTGTC-3'  Rev: 5'- TAATACGACTCACTATAGGGGTTGCTGTCGGCGACACGCTCCT-3' | [[5](#_ENREF_5)] |
| *CLIPA8*  (AGAP010731) | For: 5'- TAATACGACTCACTATAGGGAACAACGAACCCGTAGAATATG-3'  Rev: 5'- TAATACGACTCACTATAGGGGGTTAGCGCCTCGATACC-3' | [[6](#_ENREF_6)] |
| *CLIPA28*  (AGAP010730) | For: 5'-TAATACGACTCACTATAGGGAGACCACCAAGGAACCGTTCCCGCA GCAA-3'  Rev: 5'- TAATACGACTCACTATAGGGAGACCGCAACCGATGCCCCACGAT ACGAT-3' |  |
| *CLIPB4*  (AGAP003250) | For: 5'- TAATACGACTCACTATAGGGAGTAGCGGTCGTGCATCAGA-3'  Rev: 5'- TAATACGACTCACTATAGGGTGGCCTGCTAGAGCCAGCGT-3' |  |
| *CLIPB8*  (AGAP003057) | For: 5'- TAATACGACTCACTATAGGGGTCATACCGCACCCGGAGTA-3'  Rev: 5'- TAATACGACTCACTATAGGGTTCCCGTTCGACGTACGGCA-3' |  |
| *SRPN2*  (AGAP006911) | For: 5'- TAATACGACTCACTATAGGGCTGGTCAATGTGATCTACTT-3'  Rev: 5'- TAATACGACTCACTATAGGGATTGTTCCGAGGGTTTCAT-3' |  |
| *CLIPB9*  (AGAP029769) | For: 5'- TAATACGACTCACTATAGGGAATGCACGACACCGACGAGGT-3'  Rev: 5' TAATACGACTCACTATAGGGGTTTGCCCTCCTTGCGCTCA-3' |  |
| *CLIPB10*  (AGAP029770) | For: 5'- TAATACGACTCACTATAGGGGAGCGTAAGGGATGAGTTCT-3'  Rev: 5'- TAATACGACTCACTATAGGGCAGCACGTACCGTCCGTTGA-3' |  |
| *CLIPB13*  (AGAP004855) | For: 5'- TAATACGACTCACTATAGGGTACTACCGTCGCTCCGAGTA-3'  Rev: 5'- TAATACGACTCACTATAGGGTCGATGTCCGGACACAATGT-3' |  |
| *CLIPB14*  (AGAP010833) | For: 5'- TAATACGACTCACTATAGGGGACTGCAAGCAGGTCAAAGGC-3'  Rev: 5'- TAATACGACTCACTATAGGGTCCACGGAACATCTCCCGCT-3' |  |
| *CLIPB17*  (AGAP001648) | For: 5'- TAATACGACTCACTATAGGGAGCGTGGGGAATTCCCGTGGA-3'  Rev: 5'- TAATACGACTCACTATAGGGGGATCGTCCATCAGCAGCGA-3' |  |
|  |  |  |

References

1. Habtewold T, Povelones M, Blagborough AM, Christophides GK. Transmission blocking immunity in the malaria non-vector mosquito *Anopheles quadriannulatus* species A. PLoS Pathog. 2008; 4: e1000070.

2. Povelones M, Upton LM, Sala KA, Christophides GK. Structure-function analysis of the Anopheles gambiae LRIM1/APL1C complex and its interaction with complement C3-like protein TEP1. PLoS Pathog. 2011; 7: e1002023.

3. Povelones M, Bhagavatula L, Yassine H, Tan LA, Upton LM, et al. The CLIP-Domain Serine Protease Homolog SPCLIP1 Regulates Complement Recruitment to Microbial Surfaces in the Malaria Mosquito Anopheles gambiae. PLoS Pathog. 2013; 9: e1003623.

4. Yassine H, Kamareddine L, Chamat S, Christophides GK, Osta MA. A serine protease homolog negatively regulates TEP1 consumption in systemic infections of the malaria vector Anopheles gambiae. J Innate Immun. 2014; 6: 806-818.

5. Nakhleh J, Christophides GK, Osta MA. The serine protease homolog CLIPA14 modulates the intensity of the immune response in the mosquito Anopheles gambiae. J Biol Chem. 2017; 292: 18217-18226.

6. Volz J, Muller HM, Zdanowicz A, Kafatos FC, Osta MA. A genetic module regulates the melanization response of *Anopheles* to *Plasmodium*. Cell Microbiol. 2006; 8: 1392-1405.
