# Supplementary material for: The mosquito melanization response requires hierarchical activation of non-catalytic clip domain serine protease homologs
Source: PLoS Pathog. 2019 Nov 25;15(11):e1008194. doi: 10.1371/journal.ppat.1008194 (PMC6901238; doi:10.1371/journal.ppat.1008194)
Supplement: S4 Table — (DOCX) [file ppat.1008194.s011.docx]

| **S3 Table: Primers used in qRT-PCR** | |
| --- | --- |
| Gene | Primers used for qRT-PCR |
| *CLIPB4* | For: 5'- AGTGCTGGCGCTAGAGCTG -3'  Rev: 5'- ACGAAACAGGACACTTGCC -3' |
| *CLIPB9* | For: 5'- GTCTGCCACTGACCGACTTC -3'  Rev: 5'- TTTTGCTTAACCGCGCTTCG -3' |
| *CLIPB13* | For: 5'- ACCAATTTGCTTGCCGGTTAA -3'  Rev: 5'- GCAGCAGGTCTGACAACGAT -3' |
| *CLIPB14* | For: 5'- GTTAGCAAGCGGTTTGTGCT -3'  Rev:5'- TGAAATTCCATTCCGCAACGC -3' |
| *CLIPB17* | For:5'- TGCACGACCGAAGGCATTA -3'  Rev:5'- AGCTTTTTGTGCGTGGCAC -3' |
